# Differential Responses of Rice Genotypes to Nitrogen Supply: Impacts on Nitrogen Metabolism and Chlorophyll Fluorescence Kinetics

**DOI:** 10.3390/plants14162467

**Published:** 2025-08-08

**Authors:** Zexin Qi, Wenzheng Sun, Chun Luo, Qiang Zhang, Feisal Mohamed Osman, Chenglong Guan, Ye Wang, Mengru Zhang, Xiaotong Zhang, Jiale Ding, Yuankai Zhang, Fenglou Ling, Xiaolong Liu, Zhian Zhang, Chen Xu

**Affiliations:** 1 Agronomy College, Jilin Agricultural University, Changchun 130118, China; 2 College of Life Science and Resources and Environment, Yichun University, Yichun 336000, China; 3 Institute of Agricultural Resources and Environment, Jilin Academy of Agricultural Sciences, Changchun 130033, China

**Keywords:** rice, low nitrogen tolerance, nitrogen metabolic enzymes, chlorophyll fluorescence, osmotic regulation

## Abstract

Nitrogen (N) availability significantly influences plant metabolism and productivity. The aim of this study was to assess the effects of low N stress and subsequent N supplementation on key enzymes of nitrogen metabolism, nitrogen metabolism-related substances, and chlorophyll a fluorescence kinetic parameters in rice genotypes with different nitrogen utilization efficiencies. We used the Jijing 88 (low-N tolerant) and Xinong 999 (low-N sensitive) as test materials. During the seedling, tillering, and booting stages, the 1/2N and 1/4N treatments were restored to the 1N treatment level. Nine treatments were used in this experiment: CK (1N), A1 (1/2N), A2 (1/2N restored to 1N during the seedling stage), A3 (1/2N restored to 1N during the tillering stage), A4 (1/2N restored to 1N during the booting stage), B1 (1/4N), B2 (1/4N restored to 1N during the seedling stage), B3 (1/4N restored to 1N during the tillering stage), and B4 (1/4N restored to 1N during the booting stage). Key physiological responses, nitrogen compounds, enzymes activities, and chlorophyll a fluorescence kinetics were analyzed. Under low nitrogen conditions, the growth and nitrogen assimilation of rice were inhibited. Compared to XN 999, JJ 88 maintains higher levels of dry matter, nitrate reductase activity (NR), glutamine synthetase activity (GS), glutamate oxaloacetate transaminase activity (GOT), glutamate pyruvate transaminase activity (GPT), as well as nitrate (NO_3_^−^) and ammonium (NH_4_^+^) nitrogen contents. After N supplementation during the early growth stage, both JJ 88 and XN 999 exhibit recovery capabilities. However, in the late growth stage, JJ 88 demonstrates superior recovery capabilities. In addition to enhancing nitrogen metabolism levels, there is also an increase in the content of osmotic regulation substances such as soluble sugars, free amino acids, and proline, along with responses in chlorophyll fluorescence kinetic parameters. This was primarily manifested in the enhancement of performance index (PI_ABS_, PI_total_), and quantum yield (φ_EO_, φ_RO_, ψ_EO_), which maintain photosynthetic performance and electron transport efficiency. The research findings indicated that reducing N supply during the early growth stage and restoring N levels in the later stage are beneficial for the recovery of low-nitrogen-tolerant rice varieties. Therefore, in the context of sustainable agricultural production, the breeding of low-nitrogen-tolerant rice varieties and the optimization of N fertilizer management are crucial.

## 1. Introduction

Rice (*Oryza sativa* L.) is a staple food crop and serves as the primary source of sustenance for more than fifty percent of the global population [1,2]. Over the past fifty years, rice production has risen in tandem with global population growth, driven by the development of high-yielding varieties and the use of chemical fertilizers [3]. N is a vital nutrient for the growth and development of rice. However, N supply in agricultural soils is often inadequate to support optimal rice yields. Consequently, the application of additional N fertilizer is a crucial strategy for enhancing rice production [4]. Global N fertilizer consumption has surpassed 110 million tons annually. In China, the average application rate of N fertilizer has reached 180 kg/hm^2^, 75% higher than the global average. However, their nitrogen use efficiency (NUE) is only between 28% and 35%, 15% to 20% lower than the global average NUE [5,6,7]. It is estimated that at least fifty percent of the N fertilizer applied to rice fields is not utilized by the plants. This inefficiency leads to significant N leaching, which exacerbates soil acidification, contributes to eutrophication, increases greenhouse gas emissions, and results in environmental pollution. Consequently, these issues may trigger broader challenges, including global economic instability and climate change [7]. In addition to reducing N fertilizer application and utilizing high-yield, efficient cultivation techniques, breeding nitrogen-efficient rice varieties is also a primary research goal aimed at minimizing N fertilizer losses [8]. Breeding low-nitrogen-tolerant rice varieties can help reduce production costs and the environmental pollution associated with N fertilizer loss in the field, while simultaneously maintaining rice yields.

The process of photosynthesis involves a large number of proteins that together account for the majority of the nitrogen in leaves [9]. Chlorophyll fluorescence kinetics is a rapid and nonintrusive technique for investigating photosynthesis, as it can swiftly indicate damage to a photosynthetic apparatus [10,11]. Photosystem II (PSII) is highly sensitive to stress. Chlorophyll fluorescence analysis is a valuable tool for understanding the various locations and degrees of stress damage to a plant’s photosynthetic machinery. The N application rate is closely related to gas exchange and chlorophyll fluorescence efficiency. Under N deficiency stress, a reduction in the content of the photosynthetic pigments in plants weakens the chloroplasts’ ability to absorb light, decreases the rate of photosynthetic electron transfer, and lowers the efficiency of light energy utilization. Consequently, this limits photosynthetic efficiency and impairs plant growth [12]. Studies have shown that the activity of the photosynthetic system and the efficiency of electron transport in peanuts decrease with increasing nitrogen. Optimizing nitrogen application enhances the activity of the PSII reaction center, facilitates electron transport in the photosynthetic electron chain, reduces energy dissipation in leaves, and is beneficial for increasing yield [13]. Furthermore, studies have shown that an increase of N supply can alleviate the negative effects induced by salinity stress and improved plant growth by maintaining the integrity of the photosynthesis and chlorophyll fluorescence processes of oat plants [14]. Therefore, the impact of changes in N supply on the photosynthetic performance of rice with different nitrogen efficiencies may also vary.

Chlorophyll fluorescence kinetics (OJIP) is an ultra-sensitive, reliable, rapid, and straightforward measurement method that is widely employed to study the effects of nutrients on plants. A detailed analysis of OJIP kinetics yields valuable data regarding the functioning of the photosynthetic apparatus [15]. Research on ryegrass found a higher accumulation of reduced plastoquinone A (QA^−^) and a higher rate of QA deoxidation under medium and lower N concentrations, indicating a higher electron transport rate between QA and QB [16]. Kaltrina et al. [17] observed that under an insufficient N supply, plants exhibit signs of nutritional stress, such as a faster reduction of the primary acceptor, a decreased ability of photosystem II donor sites, and lower performance indicators. Different N supply levels impact photosynthetic metabolism in various ways, depending on the plant species and its phenological stage. As leaf nutrient requirements transition from a sink to a source, the JI stage of the OJIP curve during the vegetative phase exhibits a downward trend, whereas during the reproductive phase, it displays an upward trend [18].

Insufficient N availability during any growth period will inhibit the growth, development, and metabolic levels of rice, leading to reduced yields [19,20]. A low N supply during the tillering and heading stages will lead to slow growth, a reduced tiller number, poor grain filling, and a reduced yield [20,21]. Chen et al. [22] have shown that double-season super hybrid rice is more sensitive to N deficiency during the tillering stage, indicating that the panicle differentiation stage is an effective compensation period. Other studies have demonstrated that the N stress memory experienced by seedlings may also influence their subsequent responses during later stages of nutrition [23]. Zhang et al. [24] demonstrated that excessive N fertilization results in delayed flowering times and maturity, thereby illustrating low resource use efficiency. Currently, research on the reduction of N fertilizer application during the early stages of rice cultivation and its subsequent effects on nitrogen metabolism and photosynthetic performance following N fertilizer recovery remains limited. This study aimed to investigate the effects of N recovery at various phenological phases on nitrogen metabolism and chlorophyll fluorescence kinetics in rice genotypes with differing nitrogen use efficiencies. This experiment utilized low-nitrogen-tolerant and nitrogen-sensitive rice varieties as materials, employing hydroponics throughout the entire growth period. It explored the effects of nitrogen deficiency and compensation at different phenological phases on the accumulation of dry matter, nitrogen-containing substances, osmotic substances, key enzymes involved in nitrogen metabolism, and chlorophyll fluorescence kinetics. This provides a theoretical basis for the cultivation of rice varieties under low nitrogen conditions and the efficient utilization of nitrogen fertilizers.

## 2. Results

### 2.1. Dry Matter Accumulation

**Figure 1:**
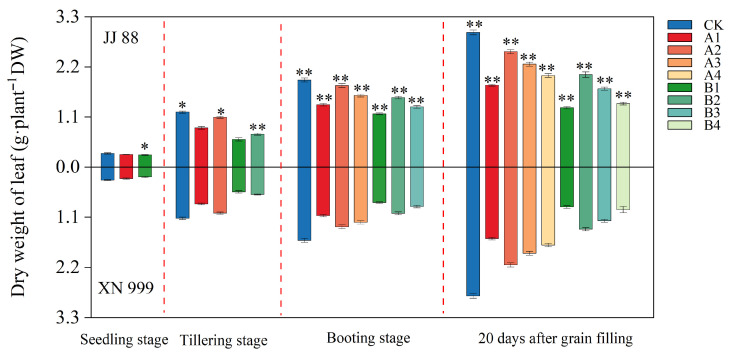
Effect of N concentration on leaf dry matter accumulation of JJ 88 and XN 999. Values are presented as means ± SD, n = 3. Different letters on the column represent significant differences (*p* < 0.05) between different treatments of the same rice varieties based on Duncan’s test. The significance of differences between JJ 88 and XN 999 during the same periods and treatments are indicated with asterisks, and were obtained by two-tailed Student’s *t*-test: *, *p* < 0.05; **, *p* < 0.01.

Leaf dry matter accumulation showed an increasing trend with time (Figure 1). After the N supply level was increased, the dry weight of the rice leaves increased, but the changes were different between JJ 88 and XN 999. At the booting stage and 20 days after grain filling, except for under the CK treatment, the leaf dry weight under all other treatments for JJ 88 was significantly (*p* < 0.01) higher than that of XN 999. At the booting stage, compared with the CK treatment, the leaf dry weight of JJ 88 under the A1, A2, A3, B1, B2, and B3 treatments decreased by 28.37%, 6.40%, 17.76%, 38.84%, 20.13%, and 30.81%, respectively; the leaf dry weight of XN 999 under the A1, A2, A3, B1, B2, and B3 treatments decreased by 33.70%, 18.44%, 24.65%, 51.38%, 36.88%, and 46.14%, respectively. Twenty days after grain filling, compared with the CK treatment, the leaf dry weight of JJ 88 under the A1, A2, A3, A4, B1, B2, B3, and B4 treatments decreased by 39.20%, 14.28%, 23.55%, 32.05%, 55.89%, 31.31%, 41.77%, and 52.83%, respectively; the leaf dry weight of XN 999 under the A1, A2, A3, A4, B1, B2, B3, and B4 treatments decreased by 44.47%, 24.12%, 33.08%, 39.45%, 69.05%, 51.78%, 58.27%, and 67.02%, respectively.

### 2.2. Nitrogenous Compounds

**Figure 2:**
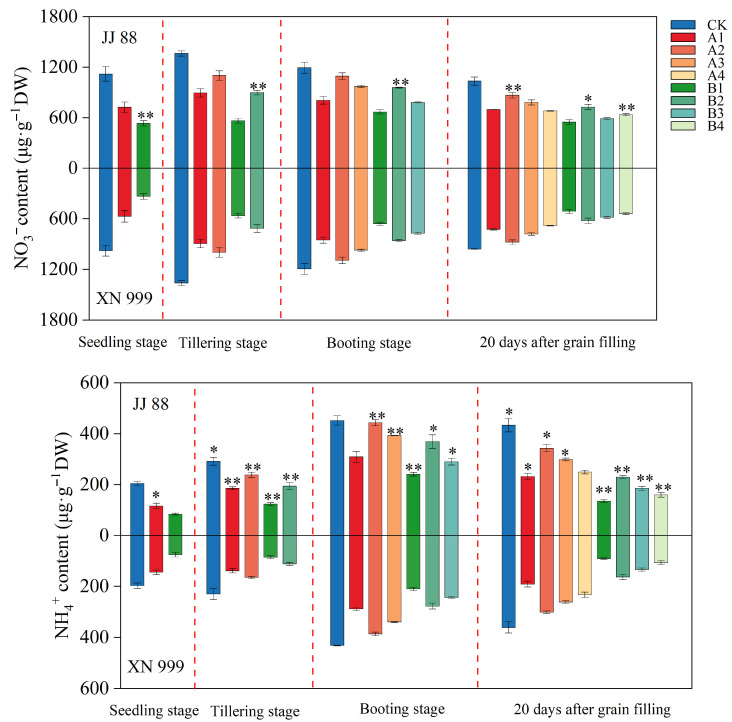
Effect of nitrogen concentration on NO_3_^−^ and NH_4_^+^ contents of JJ 88 and XN 999. Values are presented as means ± SD, n = 3. The significance of differences between JJ 88 and XN 999 during the same periods and treatments are indicated with asterisks, and the values were obtained by two-tailed Student’s *t*-test: *, *p* < 0.05; **, *p* < 0.01.

From Figure 2, it can be seen that the NO_3_^−^ and NH_4_^+^ contents showed a trend of increasing and then decreasing. At the booting stage, the NO_3_^−^ content of JJ 88 under the B2 treatment was significantly (*p* < 0.01) higher than that of XN 999, and it was also significantly (*p* < 0.05) higher than XN 999 20 days after grain filling. At the booting stage and 20 days after grain filling, under the treatments of A2, A3, B1, B2, and B3, the NH_4_^+^ content of JJ88 consistently remained significantly (*p* < 0.05) higher than that of XN999. At the booting stage, compared with the CK treatment, the NH_4_^+^ content of JJ 88 under the A1, A2, A3, B1, B2, and B3 treatments decreased by 31.68%, 2.00%, 13.23%, 46.73%, 18.39%, and 35.92%, respectively; the NH_4_^+^ content of XN 999 under the A1, A2, A3, B1, B2, and B3 treatments decreased by 33.24%, 10.53%, 21.36%, 51.41%, 35.81%, and 43.53%, respectively. Twenty days after grain filling, compared with the CK treatment, the NH_4_^+^ content of JJ 88 under the A1, A2, A3, A4, B1, B2, B3, and B4 treatments decreased by 46.49%, 20.89%, 31.09%, 42.49%, 68.91%, 47.03%, 57.17%, and 63.25%, respectively; the NH_4_^+^ content of XN 999 under the A1, A2, A3, A4, B1, B2, B3, and B4 treatments decreased by 47.09%, 16.65%, 27.51%, 35.59%, 74.79%, 54.94%, 63.04%, and 70.53%, respectively.

### 2.3. Soluble Sugar Content, Amino Acid and Proline Content

After low N treatment, the content of soluble sugars and free amino acids decreased, while the content of proline increased (Table S1). At the booting stage and 20 days after grain filling, the soluble sugar and free amino acid contents of JJ 88 were consistently significantly higher (*p* < 0.05) than those of XN 999 under all other treatments except for CK and A1. Under the treatments of B2 and B3, the proline content of XN 999 was significantly (*p* < 0.01) higher than that of JJ88. At the booting stage, compared to the CK treatment, the decrease in soluble sugar content for JJ 88 ranged from 5.67% to 33.58%, while for XN 999, it ranged from 17.46% to 56.22%. The decrease in free amino acid content for JJ 88 ranged from 9.78% to 51.45%, while for XN 999, it ranged from 21.35% to 56.40%. Twenty days after grain filling, compared to the CK treatment, the decrease in soluble sugar content for JJ 88 ranged from 18.07% to 57.75%, while for XN 999, it ranged from 31.47% to 71.02%. The decrease in free amino acid content for JJ 88 ranged from 17.36% to 53.25%, while for XN 999, it ranged from 17.84% to 58.24%. Among them, compared with the CK treatment, the increase in proline content for JJ 88 ranged from 19.57% to 140.53%, and for XN 999, it ranged from 25.54% to 178.13%.

### 2.4. NR and Gs Activity

**Figure 3:**
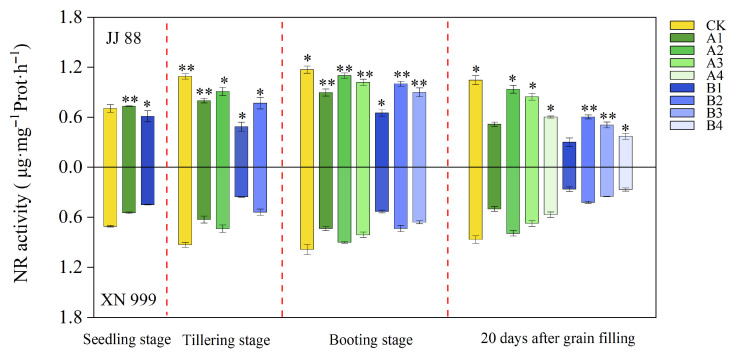

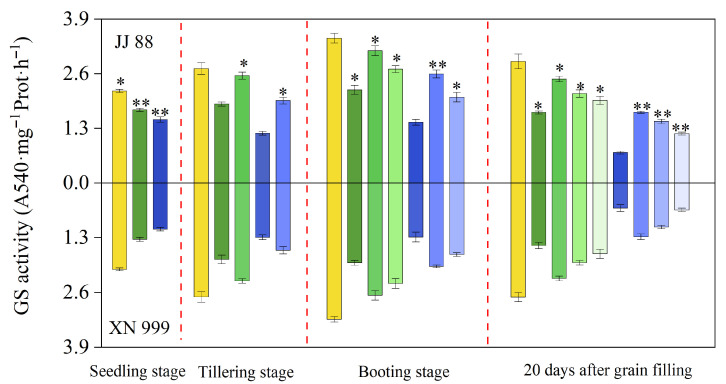
Effect of N concentration on the NR and GS activities of JJ 88 and XN 999. Values are presented as means ± SD, n = 3. The significance of differences between JJ 88 and XN 999 during the same periods and treatments are indicated with asterisks and were obtained by two-tailed Student’s *t*-test: *, *p* < 0.05; **, *p* < 0.01.

As shown in Figure 3, low N treatment reduced rice NR and GS activities. The activities of NR and GS increased with the increase of the N supply level, and the changes of JJ 88 and XN 999 were different. At the booting stage, the NR and GS activities of JJ 88 under the A1, A2, A3, B1, and B2 treatments were consistently significantly (*p* < 0.05) higher than those of XN 999. Twenty days after grain filling, the NR and GS activities of JJ 88 under the A2, A3, A4, B2, B3, and B4 treatments were consistently significantly (*p* < 0.05) higher than those of XN 999. At the booting stage, compared to the CK treatment, the decrease in NR activity for JJ 88 ranged from 6.21% to 44.30%, while for XN 999, it ranged from 8.70% to 46.16%. The decrease in GS activity for JJ 88 ranged from 8.63% to 57.95%, while for XN 999, it ranged from 17.60% to 60.40%. Twenty days after grain filling, compared to the CK treatment, the decrease in NR activity for JJ 88 ranged from 10.49% to 65.15%, while for XN 999, it ranged from 10.56% to 69.02%. The decrease in GS activity for JJ 88 ranged from 14.50% to 74.90%, while for XN 999, it ranged from 16.42% to 76.44%.

### 2.5. GOT and GPT Activity

**Figure 4:**
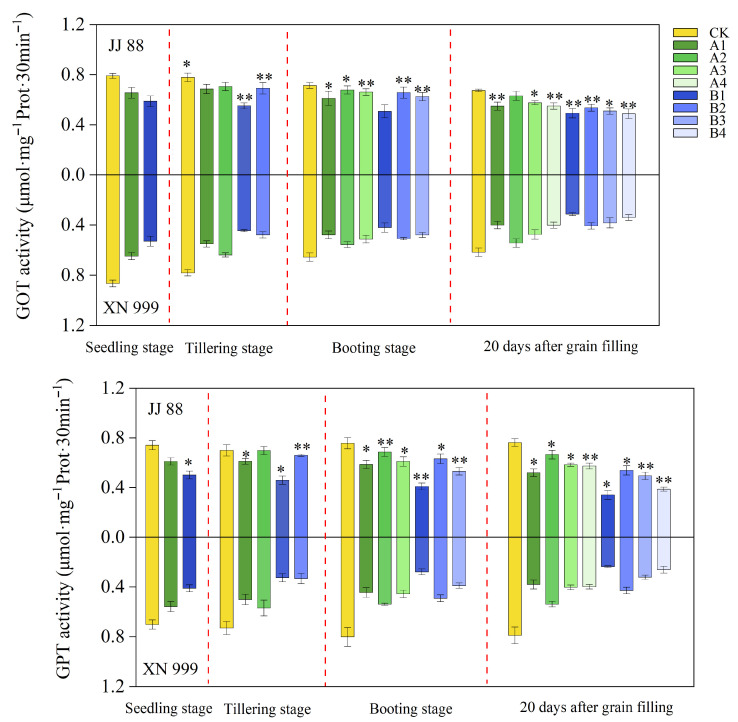
Effect of N concentration on GOT and GPT activities of JJ 88 and XN 999. Values are presented as means ± SD, n = 3. The significance of differences between JJ 88 and XN 999 during the same periods and treatments are indicated with asterisks and were obtained by two-tailed Student’s *t*-test: *, *p* < 0.05; **, *p* < 0.01.

As seen from Figure 4, the activities of GOT and GPT tended to change at a stable rate during the reproductive period. The activities of GOT and GPT decreased slightly after low N treatment. After the N supply level increased, the activity of JJ 88’s and XN 999’s GOT increased slightly, but the increase in GPT activity was relatively large. At the booting stage, the GOT and GPT activities of JJ 88 under the A1, A2, A3, B2, and B3 treatments were consistently significantly (*p* < 0.05) higher than those of XN 999. Twenty days after grain filling, the GOT and GPT activities of JJ 88 under the A1, A3, A4, B1, B2, B3, and B4 treatments were consistently significantly (*p* < 0.05) higher than those of XN 999. At the booting stage, compared to the CK treatment, the decrease in GOT activity for JJ 88 ranged from 4.96% to 28.88%, while for XN 999, it ranged from 15.18% to 35.97%. The decrease in GPT activity for JJ 88 ranged from 9.27% to 45.87%, while for XN 999, it ranged from 32.80% to 65.34%. Twenty days after grain filling, compared to the CK treatment, the decrease in GOT activity for JJ 88 ranged from 6.54% to 27.66%, while for XN 999, it ranged from 11.94% to 49.30%. The decrease in GPT activity for JJ 88 ranged from 12.76% to 55.33%, while for XN 999, it ranged from 31.91% to 70.15%.

### 2.6. Chlorophyll Fluorescence Related Parameters

After low N treatment, Mo, φ_Do_, and δ_Ro_ increased, Sm, RC/ABS, γRC, PI_ABS_, PI_total_, φ_Po_, φ_Eo_, φ_Ro_, and ψ_Eo_ decreased (Table S2). At the booting stage and 20 days after grain filling, JJ 88 consistently showed significantly (*p* < 0.05) higher PI_ABS_, PI_total_, φ_Eo_, and ψ_Eo_ values compared to XN 999 under most treatments, while XN 999 showed significantly (*p* < 0.05) higher Mo and δ_Ro_ values than JJ 88 in most treatments. At the booting stage, compared to the CK treatment, the increase in Mo for JJ 88 ranged from 10.11% to 46.05%, while for XN 999, it ranged from 8.31% to 41.86%; the decrease in PI_ABS_ for JJ 88 ranged from 20.82% to 75.29%, while for XN 999, it ranged from 27.85% to 84.20%. The decrease in PI_total_ for JJ 88 ranged from 9.79% to 57.96%, while for XN 999, it ranged from 28.76% to 73.26%; the decrease in φ_Eo_ for JJ 88 ranged from 6.07% to 45.54%, while for XN 999, it ranged from 9.30% to 59.94%. The decrease in ψ_Eo_ for JJ 88 ranged from 4.76% to 40.52%, while for XN 999, it ranged from 6.97% to 54.29%; the increase in δ_Ro_ for JJ 88 ranged from 2.04% to 26.53%, while for XN 999, it ranged from 1.37% to 29.92%. Twenty days after grain filling, compared to the CK treatment, the increase in Mo for JJ 88 ranged from 1.00% to 43.52%, while for XN 999, it ranged from 5.58% to 44.17%; the decrease in PI_ABS_ for JJ 88 ranged from 16.78% to 73.48%, while for XN 999, it ranged from 24.39% to 87.15%. The decrease in PI_total_ for JJ 88 ranged from 7.91% to 61.24%, while for XN 999, it ranged from 28.01% to 75.49%; the decrease in φ_Eo_ for JJ 88 ranged from 4.26% to 37.13%, while for XN 999, it ranged from 6.69% to 62.60%. The decrease in ψ_Eo_ for JJ 88 ranged from 1.65% to 28.36%, while for XN 999, it ranged from 3.53% to 55.13%; the increase in δ_Ro_ for JJ 88 ranged from 4.65% to 23.55%, while for XN 999, it ranged from 3.70% to 34.13%.

### 2.7. Redundancy Analysis

**Figure 5:**
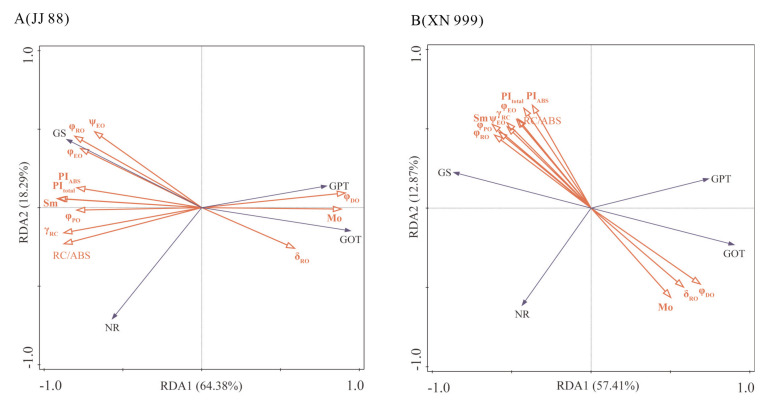
Redundancy analysis of nitrogen metabolism enzymes and chlorophyll fluorescence-related parameters in JJ88 (**A**) and XN999 (**B**).

As can be seen from Figure 5, the two axes RDA1 and RDA2 explain 64.38% and 18.29%, respectively, of the response characteristics of JJ 88 nitrogen metabolism enzymes to chlorophyll fluorescence characteristics, and explain 57.41% and 12.87% of the response characteristics of XN 999 nitrogen metabolism enzymes to chlorophyll fluorescence characteristics. This showed that the RDA results were reliable and that rice nitrogen metabolism enzymes were closely related to the chlorophyll fluorescence characteristics. The NR and GS of JJ 88 were positively correlated with φ_PO_, φ_EO_, Sm, RC/ABS, PI_ABS_, PI_total_, and γRC. The GS of XN 999 was positively correlated with φ_PO_, φ_EO_, φ_RO_, γRC, ψ_EO_, Sm, RC/ABS, PI_ABS_, and PI_total_.

## 3. Discussion

N is an essential element in the growth and development of plants, playing a critical regulatory role in their physiological processes. In nature, the inorganic N available to plants is mainly NO_3_^−^ and NH_4_^+^. This study demonstrated that NO_3_^−^ content decreased under low N conditions but increased with higher N supply levels. The research indicated that low N treatment led to a reduction in NO_3_^−^ content, whereas an increase in N supply resulted in elevated NO_3_^−^ levels. Notably, compared to XN 999, the NO_3_^−^ content of JJ 88 exhibited a significant increasing trend. This suggests that the primary N assimilation in rice leaves plays a crucial role in regulating the plant’s adaptation to varying N levels, with JJ 88 leaves showing greater potential for N assimilation capacity. On the other hand, as the NO_3_^−^ content in leaves increases, increasing the stomatal aperture is beneficial to gas exchange, thus increasing the stomatal conductance of the leaves [25]. NR is the rate-limiting enzyme for NO_3_^−^ assimilation. NO_3_^−^ assimilation is a highly energy-consuming process [26]. Due to nitrate-induced nitrate reductase, the nitrate uptake rate at the induction site is the main controlling factor for NR activity levels [27]. This study demonstrated that the NR activity in rice leaves decreased following a reduction in nitrate NO_3_^−^ levels. Upon N resupply, both NR activity and NO_3_^−^ content exhibited an increase. This phenomenon may be explained by the compartmentalization of NO_3_^−^ assimilation in leaves, as NO_3_^−^ is primarily stored in vacuoles, which are distinct from the NR located in cytoplasm. It is also possible that the N supply improved the photosynthetic activity of the leaves, thereby increasing the absorption of NO_3_^−^. The varying responses of NO_3_^−^ in the rice varieties JJ 88 and XN 999 to changes in N supply levels highlight the differences in the role of NO_3_^−^ reduction in N adaptation among rice cultivars. Therefore, varieties with a low nitrogen tolerance possess strong NR activity and NO_3_^−^ content. This capability can be maintained even during the later stages of growth.

The assimilation of NH_4_^+^ consumes energy, which may reduce the energy available for photosynthesis, mitochondrial respiration, and other cytoplasmic reactions, depending on the site of the reaction. Enhanced nitrogen assimilation in leaves under stress conditions contributes to improved plant stress resistance, as this process utilizes the residual energy outside of photosynthesis to enhance plant adaptation to adverse conditions [28]. Following the reduction in N supply levels, the NH_4_^+^ content in rice leaves also diminished. The avoidance of excess NH_4_^+^ accumulation in plant tissues is regarded as a self-protective mechanism [29]. As a key enzyme involved in the plant assimilation of NH_4_^+^, GS is a key enzyme that combines NH_4_^+^ into amides and amino acids [30]. JJ 88 and XN 999 exhibit distinct responses to variations in N levels. Following an increase in N supply, the GS activity of JJ 88 rose significantly, surpassing that of XN 999. The accumulation of NH_4_^+^ in JJ 88 aligns with the observed trend in GS enzyme activity. With the elevated N supply, JJ 88 absorbed a greater amount of NH_4_^+^, facilitating subsequent assimilation processes. In addition, the increase in GS activity also confirmed this inference. Therefore, the accumulation of NH_4_^+^ and increased GS activity are important factors for improving rice growth under low N conditions. This suggests that low-nitrogen-tolerant rice varieties have higher levels of GS activity and NH_4_^+^ content. After 20 days of filling, the NR activity and NH_4_^+^ content of the A2 treatment still approached the level of the CK treatment. In addition, the ability of low-nitrogen-tolerant rice to absorb NH_4_^+^ may also be related to the activity of phosphoenolpyruvate carboxylase (PEPC). PEPC is as anaplerotic enzyme used to load a tricarboxylic acid (TCA) cycle with carbon skeletons that compensate the intermediates diverted for biomolecule synthesis such as amino acids. A plant’s excessive uptake of NH_4_^+^ often provokes a stress situation. When plants face NH_4_^+^ stress, N assimilation is greatly induced and thus, requires the supply of carbon skeletons coming from the TCA cycle [31]. Inorganic nitrogen can be absorbed and utilized only after it is assimilated into organic nitrogen, among which glutamic acid and glutamine are the most important anabolic metabolites for ammonia synthesis [32]. GOT is the most active aminotransferase, facilitating the conversion of α-ketoglutarate and aspartate to oxaloacetate and glutamate, thereby generating aspartic acid, a precursor of the aspartate family of amino acids. GPT is a crucial enzyme in the nitrogen metabolism pathway, catalyzing the production of alanine. Additionally, aspartate (Asp) serves as a detoxification molecule for NH_4_^+^ and acts as a precursor for the synthesis of branched-chain amino acids (BCAAs). BCAAs, which are derived from the Asp pathway, include methionine (Met), threonine (Thr), and isoleucine (Ile), and these amino acids provide essential precursors for various plant secondary metabolites [33]. For example, ethylene synthesis, which plays an important role in plant stress responses, is derived from Met. Following the increase in N supply levels, the activities of glutamate oxaloacetate transaminase (GOT) and glutamate pyruvate transaminase (GPT) in the leaves of various rice varieties were found to increase. This suggests that the enhancement of methionine (Met) may play a crucial role in improving the photosynthetic capacity of rice leaves under low N conditions. Furthermore, N levels exhibited a consistent trend in relation to the activities of GOT and GPT enzymes across different rice varieties that are tolerant of low N conditions. However, the GOT and GPT activities in the low-nitrogen-tolerant rice varieties remained consistently high.

Under abiotic stress conditions, nitrogen assimilation products, including sugars and amino acids, play a crucial role in adapting to environmental changes as osmotic regulatory substances [34]. Studies have demonstrated that amino acids can act as osmotic agents under stress conditions, and increased synthesis enhances plant adaptability [35]. In this study, we observed a decrease in the amino acid content of rice leaves under low N conditions, followed by an increase upon elevating the N supply level. Notably, the free amino acid content of JJ 88 exhibited a more pronounced increase compared to XN 999 after the N supply was enhanced. These variations in free amino acid content align with changes in GS activity. Among them, free amino acids provide the foundation for the formation of substances. Additionally, soluble sugar, as a direct product of plant photosynthesis, serves as the precursor for synthesizing macromolecules. In plants, soluble sugars are transported as carbohydrates and distributed across various organs, playing a crucial role in their growth and development [36]. Relevant studies have demonstrated that under conditions of stress, sugars and amino acids work synergistically to prevent cellular dehydration and to preserve both the structure and function of cell membranes [37]. Other studies have shown that the accumulation of non-structural carbohydrates such as soluble sugars under stress conditions can maintain leaf expansion [38]. This study demonstrated that low N conditions lead to a reduction in soluble sugar content, which subsequently increases with elevated N supply levels. Under stress conditions, the proline content in plants significantly increases. In this study, 20 days after filling, JJ 88 exhibited a smaller reduction in soluble sugars and a smaller increase in proline, further indicating its strong adaptability to low nitrogen conditions, as it did not require osmotic adjustment due to stress adversity.

N is a critical element in Calvin cycle enzymes and chlorophyll, in which chlorophyll content has a direct effect on photosynthetic capacity and primary production [39]. Previous studies demonstrated that N deficiency can cause the closure of stomata in leaves, blocking photosynthetic electron transfer and ultimately reducing PSII performance [40]. The kinetic curve of OJIP effectively reflects the changes in primary photochemical reactions and the photosynthetic functions of PSII. For example, hypoxic treatment induces the uncoupling of oxygen complexes. It inhibits electron transfer outside the plastic quinone pool (QA, QB), which may limit the reduction of electron acceptors at the end of a photosystem [41]. Magnesium deficiency will affect the entire photosynthetic electron transfer process of citrus seedlings, including the structural destruction of thylakoid, a loss of PSII, an inactivation of the evolved oxygen complex (OEC) and reaction center (rc), and primary quinone on the PSII receptor side. The reduction of electron acceptor (QA) and plastoquinone pools reduces the energy transfer and absorption efficiency and the transfer of electrons to dark reactions [42]. Studies have shown that limited N availability disrupts energy transfer within PSII. This disruption occurs due to the deactivation of reaction centers, which diminishes the efficiency of converting absorbed light energy into usable electrons [43]. This study showed that low N treatment increased Mo, φ_DO_, and δ_RO_, and decreased Sm, RC/ABS, γRC, PI_ABS_, PI_total_, φ_PO_, φ_EO_, φ_RO_, and ψ_EO_. As the N supply level increases, the amplitude of the increases and decreases in related parameters decreases. As the N supply level increases, the amplitude of the increases and reductions of associated parameters decreases. This showed that low N treatment harms the PSII system, and increasing the N supply level has a positive effect on the PSII system. However, the changes in the chlorophyll fluorescence-related parameters of JJ 88 and XN 999 are different. Their main performance indicated the following: 20 days after filling, the values for PI_ABS_, PI_total_, φ_EO_ φ_RO_, and ψ_EO_ of JJ88 remain higher than those of XN999. PI_ABS_ as electrons describing the reduction of photons absorbed into the final PSI receptor. PI_total_ is a performance indicator describing the function of the electron transport chain. The PSII is the functional response of the electronic transfer center between systems or the reduction of PSI electron acceptors [44]. The results indicate that an increase in N supply levels enhances the protective role of JJ 88 on the photosynthetic mechanism, improves light energy conversion efficiency, and optimizes electron transfer efficiency. φ_PO_, φ_EO_, ψ_EO_, φ_RO_, and δ_RO_ represent quantum efficiency or the flux ratio. In this study, φ_PO_ increased with increasing N levels. This may be attributed to the role of N in the electron transport chain, which enhances the efficiency of electron acceptors and promotes electron transfer between PSI and PSII. Furthermore, ψ_EO_ reflects the possibility of electrons moving beyond QA^−^. This showed that low N treatment reduces the excitation pressure of PSII (ψ_EO_), while an increase in N concentration increases the excitation pressure of PSII. In this study, low N treatment reduced φ_RO_. This showed that the terminal electron acceptor pool on the PSI receptor side decreased after low N treatment. Studies have shown that a reduction of φ_RO_ is an essential sign of a decrease in PSI content [15]. In summary, rice with a strong low-nitrogen tolerance can still maintain photosynthetic performance and photosynthetic electron transport capacity during the late growth stage.

## 4. Materials and Methods

### 4.1. Plant Materials

Rice seeds of Jijing 88 (JJ 88), characterized by low nitrogen tolerance, and Xinong 999 (XN 999), known for its sensitivity to low N conditions, were obtained from the Rice Research Institute of Jilin Agricultural University. The screening method of low-nitrogen-tolerant rice varieties can be found in Qi et al. [45].

### 4.2. Experimental Design

Previous studies have shown that N affects the antioxidant system of rice with different low nitrogen tolerances, but have not examined changes in nitrogen metabolism and the fluorescence levels of rice after N restoration during the critical reproductive period of the plant [45]. The experiment was conducted in 2023, under natural light, in a greenhouse located at Jilin Agricultural University. The seeds were disinfected with 0.5% sodium hypochlorite for 10 min. After disinfection, the residual sodium hypochlorite was removed from the surface of the rice seeds using distilled water. The seeds were subsequently placed in culture dishes for germination. The germinated seeds were then placed in a seedling tray filled with vermiculite, with three seeds per hole. The seedling tray was kept in water for five days. Next, the seedlings were transferred to a half-concentration Kimura B nutrient solution for seven days. Subsequently, the half-concentration Kimura B solution was replaced with an improved Kimura B formulation and incubated for 12 days, which included NO_3_^−^ and NH_4_^+^ as N sources for the N treatments. Three N concentrations were used: 1N: 1.6 mM NO_3_^−^ and 1.6 mM NH_4_^+^; 1/2N: 0.8 mM NO_3_^−^ and 0.8 mM NH_4_^+^; and 1/4N: 0.4 mM NO_3_^−^ and 0.4 mM NH_4_^+^. Based on a comprehensive review of the relevant literature, preliminary experiments on N concentration were conducted, thereby determining NO_3_^−^ and NH_4_^+^ as nitrogen sources and setting the treatment levels [46].

**Figure 6:**
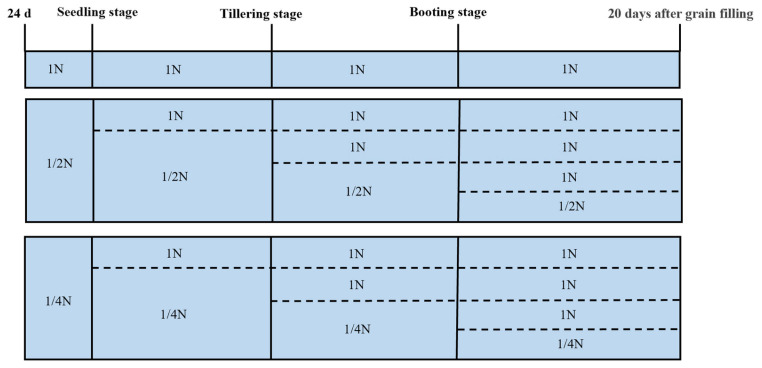
Schematic diagram of processing in different periods.

Three replications were set up for each treatment, and each replication was planted with one seedling tray (100 holes), with a size of 28 cm × 54 cm. For the recovery study, the 1/2N and 1/4N solutions were replaced with the 1N solution after rice was grown to 24 days of the seedling stage and after tillering and spike initiation, as shown in Figure 6. Measurements and sampling were carried out on the 45th day after sowing (seedling stage), at the tillering (60th day after sowing) and booting stages (80th day after sowing), and 20 days after grain filling (110th day after sowing). There were a total of nine treatments in this experiment: CK (1N supply), A1 (1/2N supply), A2 (1/2N supply restored to 1N supply during the seedling stage), A3 (1/2N supply restored to 1N supply during the tillering stage), A4 (1/2N supply restored to 1N supply during the booting stage), B1 (1/4N supply), B2 (1/4N supply restored to 1N supply during the seedling stage), B3 (1/4N supply restored to 1N supply during the tillering stage), and B4 (1/4N supply restored to 1N supply during the booting stage). The nutrient solution was replaced every three days and adjusted to pH 5.5 using HCl or NaOH, depending on the initial pH. The contents of other elements in the nutrient solution are shown in Table S3.

### 4.3. Measurement Indicators and Methods

#### 4.3.1. Chlorophyll a Fluorescence Induction Kinetics (OJIP) Analysis

Dry weight determination: After the seedling, tillering, and booting stages, and 20 days after the grain filling stage, the rice leaves were dried to a constant weight at 80 °C and weighed.

OJIP analysis: All treated rice plants were placed in a dark room at the same temperature for two hours. A portable photosynthesis system (Li-6800XT; LI-COR Inc., Lincoln, NE, USA) using multi-phase flash lamps was used to obtain the chlorophyll a fluorescence parameters (F_M_, F_0_, F_J_, F_I_, F_50μs_, F_300μs_), with a multiphase flash fluorometer used to study the fast fluorescence rise (OJIP) of the rice leaves. The intensity of the saturating light pulse is 15000 μmol m^−2^s^−1^, and the duration of the saturating light pulse is 1000 ms. All fluorescence parameters were calculated using Tsimilli [47], with six replicates per treatment. The calculation formulae for the chlorophyll fluorescence related parameters are as follows: M_0_ = 4 × (F_300μs_ F_50μs_)/(F_M_ − F_0_); Sm = Area/(F_M_ − F_0_); φ_Po_ = 1 − (F_0_/F_M_); φ_Eo_ = [1 − (F_0_/F_M_)] × (1 − V_J_); φ_Ro_ = [1 − (F_0_/F_M_)] × (1 − V_I_); ψ_Eo_ = 1 − V_I_; δ_Ro_ = (1 − V_I_)/(1 − V_J_); φ_Do_ = F_0_/F_M_; RC/ABS = φ_Po_ × (V_J_/M_0_); γRC = RC/(ABS + RC); PI_ABS_ = γRC/(1 − γRC) × φ_Po_/(1 − φ_Po_) × ψ_Eo_/(1 − ψ_Eo_); and PI_total_ = PI_ABS_× φ_Ro_/(1 − φ_Ro_). The OJIP response curves measured in this experiment are shown in Figure S1. This index is calculated as follows:

M_0_: the approximated initial slope of the fluorescence transient V = f (t);

Sm: the normalized total complementary area above the OJIP transient or total electron carriers per RC;

φ_Po_: the maximum quantum yield for primary photochemistry;

φ_Eo_: the quantum yield for electron transport (ET);

φ_Ro_: the quantum yield for the reduction of end electron acceptors at the PSI acceptor side (RE);

ψ_Eo_: the probability that an electron moves further than QA^−^;

δ_Ro_: the probability with which an electron from the intersystem electron carriers is transferred to reduce end electron acceptors at the PSI acceptor side (RE);

φ_Do_: the quantum yield (at t = 0) of energy dissipation;

RC/ABS: the RCs per PSII antenna Chl a;

γRC: the probability that a PSII Chl a molecule functions as an RC;

PI_ABS_: the performance index for energy conservation from the photons absorbed by PSII until the reduction of intersystem electron acceptors;

PI_total_: the performance index for energy conservation from the photons absorbed by PSII until the reduction of PSI end electron acceptors.

#### 4.3.2. Biochemistry

The nitrate nitrogen content was measured using the salicylic acid–sulfuric acid method [48]. The ammonium nitrogen content was determined by the indophenol blue colorimetric method [49]. The free amino acid content was measured using the ninhydrin staining method [50]. The soluble sugar content was determined by the anthrone–sulfuric acid method [51]. The proline content was assayed using the method described by Bates et al. [52].

#### 4.3.3. Enzyme Activity Assay

The determinations of NR and GS activities were conducted according to the methods described by Hageman and Reed [53] and Zhang et al. [54]. The activities of GOT and GPT were determined according to Wu et al. [55].

### 4.4. Statistical Analysis

Excel 2021 software was used for preliminary collation and the analysis of data. The SPSS 23.0 software was used to analyze the data. The differences in nitrogen levels among the different rice varieties were evaluated by Student’s *t*-test. Differences were considered statistically significant when *p* < 0.05. Graphs were generated using Origin 2021 software, and Canoco 5 software was used for the redundancy analysis.

## 5. Conclusions

Rice nitrogen metabolism and chlorophyll fluorescence exhibit significant sensitivity to variations in N levels. Furthermore, nitrogen metabolism plays a crucial role in enabling rice to adapt to these fluctuations in N availability. In this study, different N concentrations had varying degrees of impact on nitrogen metabolism and chlorophyll fluorescence in JJ 88 and XN 999, with the main differences in their responses occurring during the booting stage and 20 days after grain filling. Reducing N supply during the seedling stage and supplementing it later also enabled JJ 88 to recover to a level similar to that of the CK treatment, which primarily manifested in increased dry matter accumulation. Compared to XN 999, JJ 88 maintained higher activities of nitrogen metabolism enzymes (NR, GS, GOT, GPT) under low N conditions, retained its N uptake and assimilation capacity, and increased the content of NO_3_^−^ and NH_4_^+^ in the leaves. This provided a solid foundation for material synthesis, thereby increasing the content of amino acids. The smaller decrease in soluble sugars and the smaller increase in proline in JJ 88 further indicated its strong adaptability to low nitrogen conditions, as it did not require the generation of more soluble sugars and proline for osmotic adjustment. JJ 88 also demonstrated better photosynthetic performance and capability in the photosynthetic electron transport chain, mainly reflected in the positive changes in PI_ABS_, PI_total_, φ_EO_ φ_RO_, and ψ_EO_. In summary, JJ 88 exhibited strong adaptability to changes in N levels, and this adaptability was primarily manifested in the later stages of growth. However, whether low-nitrogen rice varieties can improve growth by reducing nitrogen fertilizer application in the early reproductive stage and then restoring N supply in the later stage still requires further field validation.

## Data Availability

Data are contained within the article and Supplementary Materials.

## Supplementary Materials

The following supporting information can be downloaded at https://www.mdpi.com/article/10.3390/plants14162467/s1: Figure S1: OJIP response curves; Table S1: Effect of N concentration on soluble sugar, amino acid, and proline content of JJ 88 and XN 999; Table S2: Effect of N concentration on chlorophyll fluorescence related parameters of JJ 88 and XN 999; Table S3: Composition and content in hydroponic solution.

## References

[1] Ogawa T., Oikawa S., Hirose T. (2016). Nitrogen-utilization efficiency in rice: An analysis at leaf, shoot, and whole-plant level. Plant Soil.

[2] Lee S. (2021). Recent advances on nitrogen use efficiency in rice. Agronomy.

[3] FAO (2018). Rice Market Monitor.

[4] Wang B., Zhou G., Guo S., Li X., Yuan J., Hu A. (2022). Improving nitrogen use efficiency in rice for sustainable agriculture: Strategies and future perspectives. Life.

[5] Hu B., Wang W., Chen J., Liu Y., Chu C. (2023). Genetic improvement toward nitrogen-use efficiency in rice: Lessons and perspectives. Mol. Plant.

[6] Han M., Okamoto M., Beatty P.H., Rothstein S.J., Good A.G. (2015). The genetics of nitrogen use efficiency in crop plants. Ann. Rev. Genet..

[7] Ladha J.K., Jat M.L., Stirling C.M., Chakraborty D., Pradhan P., Krupnik T.J., Sapkota T.B., Pathak H., Rana D.S., Tesfaye K. (2020). Achieving the sustainable development goals in agriculture: The crucial role of nitrogen in cereal-based systems. Adv. Agron..

[8] Neeraja C., Subramanyam D., Surekha K., Rao P.R., Rao L.S., Babu M.P., Voleti S.R. (2016). Advances in genetic basis of nitrogen use efficiency of rice. Indian J. Plant Physiol..

[9] Evans J.R., Clarke V.C. (2019). The nitrogen cost of photosynthesis. J. Exp. Bot..

[10] Zhao L.S., Li K., Wang Q.M., Song X.Y., Su H.N., Xie B.B., Zhang X.Y., Huang F., Chen X.L., Zhou B.C. (2017). Nitrogen starvation impacts the photosynthetic performance of *Porphyridium cruentum* as revealed by chlorophyll a fluorescence. Sci. Rep..

[11] Cen H., Weng H., Yao J., He M., Lv J., Hua S., Li H.Y., He Y. (2017). Chlorophyll fluorescence imaging uncovers photosynthetic fingerprint of citrus Huanglongbing. Front. Plant Sci..

[12] Zhong C., Cao X., Bai Z., Zhang J., Zhu L., Huang J., Jin Q. (2018). Nitrogen metabolism correlates with the acclimation of photosynthesis to short-term water stress in rice (*Oryza sativa* L.). Plant Physiol. Biochem..

[13] Guo P., Ren J., Shi X., Xu A., Zhang P., Guo F., Feng Y., Zhao X., Yu H., Jiang C. (2024). Optimized nitrogen application ameliorates the photosynthetic performance and yield potential in peanuts as revealed by OJIP chlorophyll fluorescence kinetics. BMC Plant Biol..

[14] Song X., Zhou G., Ma B.-L., Wu W., Ahmad I., Zhu G., Yan W., Jiao X. (2019). Nitrogen application improved photosynthetic productivity, chlorophyll fluorescence, yield and yield components of two oat genotypes under saline conditions. Agronomy.

[15] Zhong C., Cao X., Hu J., Zhu L., Zhang J., Huang J., Jin Q. (2017). Nitrogen metabolism in adaptation of photosynthesis to water stress in rice grown under different nitrogen levels. Front. Plant Sci..

[16] Zhou C., Huang Y., Jia B., Wang Y., Wang Y., Xu Q., Li R., Wang S., Dou F. (2018). Effects of cultivar, nitrogen rate, and planting density on rice-grain quality. Agronomy.

[17] Kaltrina R., Kristi B., Dea Z., Lulezim S., René H., Jakob S., Reinhard B. (2020). Alpine ecology, plant biodiversity and photosynthetic performance of marker plants in a nitrogen gradient induced by Alnus bushes. BMC Ecol..

[18] Shao A., Sun Z., Fan S., Xu X., Wang W., Amombo E., Yin Y., Li X., Wang G., Wang H. (2020). Moderately low nitrogen application mitigate the negative effects of salt stress on annual ryegrass seedlings. PeerJ.

[19] Zhang J., Wan L., Igathinathane C., Zhang Z., Guo Y., Sun D., Cen H. (2021). Spatiotemporal heterogeneity of chlorophyll content and fluorescence response within rice (*Oryza sativa* L.) canopies under different nitrogen treatments. Front. Plant Sci..

[20] Zhao M., Li J., Zhang B., Dong Z., Wang M. (2006). The compensatory mechanism in exploring crop production potential. Acta Agron. Sin..

[21] Tsukaguchi T., Taniguchi Y., Ito R. (2016). The effects of nitrogen uptake before and after heading on grain protein content and the occurrence of basal-and back-white grains in rice (*Oryza sativa* L.). Plant Prod. Sci..

[22] Chen X., Huang L., Zhong L., Huang W., He H. (2015). Nitrogen deficiency and compensatory effects in super hybrid late rice during different growth and development stages. Acta Agric. Univ. Jiangxiensis.

[23] Xiong Q., Tang G., Zhong L., He H., Chen X. (2018). Response to nitrogen deficiency and compensation on physiological characteristics, yield formation, and nitrogen utilization of rice. Front. Plant Sci..

[24] Zhang S., Zhang Y., Li K., Yan M., Zhang J., Yu M., Tang S., Wang L., Qu H., Luo L. (2021). Nitrogen mediates flowering time and nitrogen use efficiency via floral regulators in rice. Curr. Biol..

[25] Li P., Weng J., Zhang Q., Yu L., Yao Q., Chang L., Niu Q.L. (2018). Physiological and biochemical responses of *Cucumis melo* L. chloroplasts to low-phosphate stress. Front. Plant Sci..

[26] Forde B.G., Clarkson D.T. (1999). Nitrate and ammonium nutrition of plants: Physiological and molecular perspectives. Adv. Bot. Res..

[27] Foyer C.H., Valadier M.H., Migge A., Becker T.W. (1998). Drought-induced effects on nitrate reductase activity and mRNA and on the coordination of nitrogen and carbon metabolism in maize leaves. Plant Physiol..

[28] Guo Y., Tan J. (2015). Recent advances in the application of chlorophyll a fluorescence from photosystem II. Photochem. Photobiol..

[29] Thu H.N.T., Shim I.S., Kobayashi K., Kenji U. (2003). Accumulation of some nitrogen compounds in response to salt stress and their relationships with salt tolerance in rice (*Oryza sativa* L.) seedlings. J. Plant Growth Regul..

[30] Konishi N., Ishiyama K., Matsuoka K., Maru I., Hayakawa T., Yamaya T., Kojima S. (2014). NADH-dependent glutamate synthase plays a crucial role in assimilating ammonium in the Arabidopsis root. Physiol. Plant..

[31] Marlon D., Théo P., Francesc M., Leyre U., Jose A., Cédric C., Izargi V., Pilar C., Ernesto I., Yves G. (2024). Natural variation in the adjustment of primary metabolism determines ammonium tolerance in the model grass *Brachypodium distachyon*. J. Exp. Bot..

[32] Joshi V., Joung J.G., Fei Z., Jander G. (2010). Interdependence of threonine, methionine and isoleucine metabolism in plants: Accumulation and transcriptional regulation under abiotic stress. Amino Acids.

[33] Ashraf M., Foolad M.R. (2007). Roles of glycine betaine and proline in improving plant abiotic stress resistance. Environ. Exp. Bot..

[34] Singh M., Singh V.P., Prasad S.M. (2016). Responses of photosynthesis, nitrogen and proline metabolism to salinity stress in *Solanum lycopersicum* under different levels of nitrogen supplementation. Plant Physiol. Biochem..

[35] Gao J., Liu L., Ma N., Yang J., Dong Z., Zhang J., Zhang J., Cai M. (2020). Effect of ammonia stress on carbon metabolism in tolerant aquatic plant—*Myriophyllum aquaticum*. Environ. Pollut..

[36] Pinheiro C., Chaves M. (2011). Photosynthesis and drought: Can we make metabolic connections from available data?. J. Exp. Bot..

[37] Fu G., Jian S., Li Y., Yue M., Xiong J., Tao L. (2010). Alterations of panicle antioxidant metabolism and carbohydrate content and pistil water potential involved in spikelet sterility in rice under water-deficit stress. Rice Sci..

[38] Li M., Xu J., Wang X., Fu H., Zhao M., Wang H., Shi L. (2018). Photosynthetic characteristics and metabolic analyses of two soybean genotypes revealed adaptive strategies to low-nitrogen stress. J. Plant Physiol..

[39] Gitelson A., Gritz Y., Merzlyak M. (2003). Relationships between leaf chlorophyll content and spectral reflectance and algorithms for nondestructive chlorophyll assessment in higher plant leaves. J. Plant Physiol..

[40] Richards R. (2000). Selectable traits to increase crop photosynthesis and yield of grain crops. J. Exp. Bot..

[41] He L., Yu L., Li B., Du N., Guo S. (2018). The effect of exogenous calcium on cucumber fruit quality, photosynthesis, chlorophyll fluorescence, and fast chlorophyll fluorescence during the fruiting period under hypoxic stress. BMC Plant Biol..

[42] Ye X., Chen X.F., Deng C.L., Yang L.T., Lai N.W., Guo J.X. (2019). Magnesium-deficiency effects on pigments, photosynthesis and photosynthetic electron transport of leaves, and nutrients of leaf blades and veins in *Citrus sinensis* seedlings. Plants.

[43] Kalaji H.M., Bąba W., Gediga K., Goltsev V., Samborska I.A., Cetner M.D., Dimitrova S., Piszcz U., Bielecki K., Karmowska K. (2018). Chlorophyll fluorescence as a tool for nutrient status identification in rapeseed plants. Photosynth. Res..

[44] Strasser R.J., Tsimilli M., Qiang S., Goltsev V. (2010). Simultaneous in vivo recording of prompt and delayed fluorescence and 820-nm reflection changes during drying and after rehydration of the resurrection plant *Haberlea rhodopensis*. Biochim. Biophys. Acta Bioenerg..

[45] Qi Z., Ling F., Jia D., Cui J., Zhang Z., Xu C., Yu L., Guan C., Wang Y., Zhang M. (2023). Effects of low nitrogen on seedling growth, photosynthetic characteristics and antioxidant system of rice varieties with different nitrogen efficiencies. Sci. Rep..

[46] Guo S., Chen G., Zhou Y., Shen Q. (2007). Ammonium nutrition increases photosynthesis rate under water stress at early development stage of rice (*Oryza sativa* L.). Plant Soil.

[47] Tsimilli-michael M. (2020). Revisiting JIP-test: An educative review on concepts, assumptions, approximations, definitions and terminology. Photosynthetica.

[48] Cataldo D.A., Haroon M., Schrader L.E., Youngs V.L. (1975). Rapid colorimetric determination of nitrate in plant tissue by nitration of salicylic acid. Commun. Soil Sci. Plant Anal..

[49] Santoni S., Bonifacio E., Zanini E. (2001). Indophenol blue colorimetric method for measuring cation exchange capacity in sandy soils. Commun. Soil Sci. Plant Anal..

[50] Yokoyama S., Hiramatsu J.I. (2003). A modified ninhydrin reagent using ascorbic acid instead of potassium cyanide. J. Biosci. Bioeng..

[51] Wang Y.Y., Khoo K.H., Chen S.T., Lin C.C., Wong C.H., Lin C.H. (2002). Studies on the immuno-modulating and antitumor activities of Ganoderma lucidum (Reishi) polysaccharides: Functional and proteomic analyses of a fucose-containing glycoprotein fraction responsible for the activities. Bioorg. Med. Chem..

[52] Bates L.S., Waldren R.P., Teare I.D. (1973). Rapid determination of free proline for water-stress studies. Plant Soil.

[53] Hageman R.H., Reed A.J. (1980). Nitrate reductase from higher plants. Meth. Enzymol..

[54] Zhang C., Peng S., Peng X., Chavez A.Q., Bennett J. (1997). Response of glutamine synthetase isoforms to nitrogen sources in rice (*Oryza sativa* L.) roots. Plant Sci..

[55] Wu L., Jiang S., Tao Q. (1998). The application of colormetric method on the determination of transaminase activity. Chin. J. Soil Sci..

