# Supplementary figures and images for: Differential Responses of Rice Genotypes to Nitrogen Supply: Impacts on Nitrogen Metabolism and Chlorophyll Fluorescence Kinetics
Source: Plants (Basel). 2025 Aug 8;14(16):2467. doi: 10.3390/plants14162467 (PMC12389245; doi:10.3390/plants14162467)

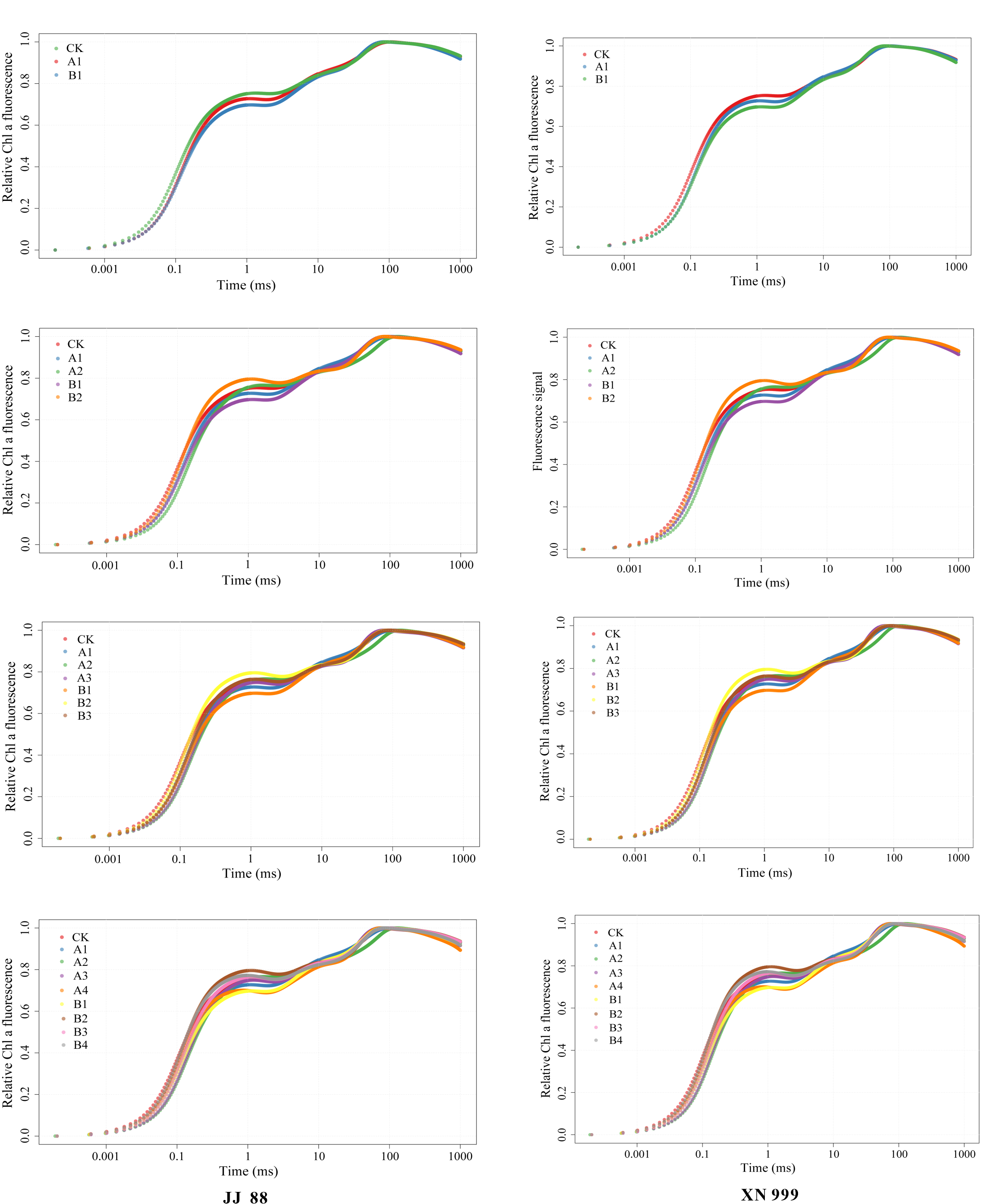

Supplement: Supplementary file 1 [file plants-14-02467-s001.zip › Supplementary File/Fig S1 OJIP response curves.tif]
